# Pharmacokinetics of Sofosbuvir and Velpatasvir for Hepatitis C Treatment in Pregnancy

**DOI:** 10.3390/biomedicines13102462

**Published:** 2025-10-10

**Authors:** Michelle L. Giles, Alexandra Dunbar, Sushena Krishnaswamy, Joe Sasadeusz, Joanne M. Said, Laura Roon, Lane R. Bushman, Kristina M. Brooks

**Affiliations:** 1 Department of Obstetrics and Gynaecology, Monash University, Melbourne, VIC 3168, Australia; 2 Department of Infectious Diseases, University of Melbourne, Melbourne, VIC 3010, Australia; 3 Department of Pharmaceutical Sciences, Skaggs School of Pharmacy and Pharmaceutical Sciences, University of Colorado Anschutz Medical Campus, Aurora, CO 80045, USA; 4 Victorian Infectious Diseases Service, Melbourne Health, Parkville, VIC 3052, Australia; 5 Department of Obstetrics, Gynaecology and Newborn Health, University of Melbourne, Parkville, VIC 3010, Australia; 6 Maternal Fetal Medicine, Joan Kirner Women’s & Children’s at Sunshine Hospital, Western Health, St Albans, VIC 3021, Australia

**Keywords:** pregnancy, antiviral therapy, hepatitis C virus, pharmacokinetics

## Abstract

**Background**: Pregnancy is a time when women are uniquely engaged with the healthcare system and are often motivated to participate in activities directed toward improvement of their own health and ensuring the health of their unborn child, which also provides an opportunity for healthcare interventions such as treatment for hepatitis C virus (HCV) infection. **Methods**: This was a multi-site, prospective, open-label, pharmacokinetic (PK) study conducted at two large maternity hospitals in Melbourne, Australia, to evaluate the safety and pharmacokinetics of antenatal sofosbuvir (SOF) and velpatasvir (VEL) treatment administered for 12 weeks during the second and third trimester. Five women were recruited and underwent detailed PK assessments across three visits. **Results**: Compared to historical data in non-pregnant women, SOF area under the concentration curve (AUC) and maximum concentrations (Cmax) were 60% and 49% higher in pregnancy, respectively. In contrast, exposure to the inactive metabolite of SOF, GS-331007, was 43% lower in pregnancy. Both Cmax and AUC for VEL in pregnancy were similar to values reported in historic non-pregnant women (~21% lower in pregnant women). SOF/VEL was safe and well tolerated. **Conclusions**: These results add to the limited published experience prescribing antivirals in pregnancy and provide further support for a larger ongoing prospective study and other efforts to support HCV treatment in pregnancy.

## 1. Introduction

Globally, an estimated 50 million people are living with hepatitis C virus (HCV), with approximately one million new infections per year [1]. Among infected people, over half will remain chronically infected unless treated with antiviral medication. Cirrhosis develops in approximately 10–20% of people after 20–30 years of chronic infection, and progression is often clinically silent; evidence of liver disease might not occur until late in the course of the disease [2]. In Australia, hepatitis C is considered a significant public health issue, and in response to this, unrestricted access to direct acting antiviral (DAA) therapy through public health subsidy was introduced in 2016 with a goal to eliminate hepatitis C as a public health threat in Australia by 2030. At the end of 2022, an estimated 60% of all people living with hepatitis C between 2016 and 2022 had been treated, although this excluded pregnant and lactating women [3].

Pregnancy is a time when women are uniquely engaged with the healthcare system and are often motivated to participate in activities directed toward improvement of their own health and ensuring the health of their unborn child. Women are recommended to attend at least eight antenatal care appointments during pregnancy [4], which provides a unique opportunity for healthcare interventions such as treatment for HCV. In this setting, and with global calls for the elimination of HCV infection, it is time to accelerate research and consider how DAAs can be integrated into the existing healthcare infrastructure of antenatal care.

Two key studies have recently shifted the paradigm for HCV treatment during pregnancy. These include the Hepatitis In Pregnancy (HIP)-1 and -2 studies. HIP-1 examined the pharmacokinetics of ledipasvir/sofosbuvir during pregnancy and found no clinically significant changes in drug exposures [5]. Treatment was started between 23 and 24 weeks’ gestation and was safe and effective with a 100% cure rate among nine women [5]. However, half of screen failures in this study were genotype 2/3 highlighting the need to evaluate a pan-genotypic regimen.

HIP-2 then enrolled women at a similar gestational age to examine sofosbuvir/velpatasvir (SOF/VEL) pharmacokinetics [6]. SOF/VEL is an orally administered, well-tolerated DAA regimen with pangenotypic activity. In published trials, a 12-week treatment course of SOF/VEL resulted in cure rates between 97 and 100% when given as a once-a-day oral pill [7]. Results on ten participants reported SOF/VEL exposures that were not clinically different in pregnancy [6]. VEL area under the curve (AUC) and SOF maximum concentration (Cmax) were similar to historical data in non-pregnant women. However, SOF area under the curve (AUC) was 38% higher while the AUC and Cmax of its major plasma metabolite, GS-331007, were 38% and 43% lower (similar to HIP-1), respectively. Despite these differences, all women with SVR12 data (n = 9) were cured, and no HCV infant transmission occurred (n = 8) [6].

Pregnancy is a time of significant physiological changes [8]. These changes can affect drug absorption, distribution, metabolism, and excretion, leading to changes in pharmacokinetics (PK). Understanding the need for dose adjustments due to these pregnancy-induced PK changes is a critical first step to ensure safe and effective administration of drugs to the mother. Here, we evaluate the PKs, safety, and efficacy of antenatal SOF/VEL treatment administered for 12 weeks during the second and third trimester in a population outside of the United States.

## 2. Materials and Methods

### 2.1. Study Design

This was a multi-site, prospective, open-label, collaborative PK study conducted at two large maternity hospitals in Melbourne, Australia. Monash Health and Sunshine Hospital are two hospitals averaging 12,000 and 6000 deliveries per year, respectively (Australian New Zealand Clinical Trial Registry 126619000054112). This study was approved by Monash Health Human Research Ethics Committee. All participants provided informed consent prior to participation.

### 2.2. Screening

As part of routine antenatal care, all pregnant women underwent a clinical review including medical history, drug and alcohol use, smoking status, and review of the laboratory investigations undertaken as per their standard medical care during pregnancy including HIV antibody testing, hepatitis B surface antigen, electrolytes, full blood examination, urinalysis, liver function tests, oral glucose tolerance test, and syphilis testing. Screening of routine booking lists identified those women who were hepatitis C antibody-positive, and these women were approached by the research team to confirm eligibility and consider participation in this study. All enrolled participants completed a baseline survey exploring their attitude to hepatitis C infection in pregnancy and to treatment for maternal and/or fetal benefit.

Treatment with SOF/VEL (sofosbuvir 400 mg/velpatasvir 100 mg (Gilead Sciences, Foster City, CA, USA) once daily) was initiated between 22 and 24 + 6 weeks’ gestation followed by three PK visits throughout the treatment course. Maternal HCV ribonucleic acid (RNA) was collected at the end of treatment and 12 weeks post treatment completion. Infants were followed through 12 months after birth.

### 2.3. Study Population

Pregnant women with HCV infection (any HCV subtype) between 18 and 45 years of age with a normal 18–22-week anomaly scan were eligible for the study. Women were excluded if they had multiple pregnancy; any abnormality on routine 18–22-week anomaly scan; negative HCV nucleic acid test; previous treatment for HCV with an NS5A inhibitor; any medications contraindicated with SOF/VEL; use of gastric acid modifiers in doses that exceeded the equivalent of 20 mg omeprazole daily or famotidine 40 mg twice daily; evidence of cirrhosis; any significant uncontrolled active or chronic cardiovascular, renal, or liver disease, or haematological, neurological, immunological or infectious disease (other than HCV); high risk of pre-term birth; co-infection with HIV-1; evidence of HBV infection (hepatitis B surface antigen (HBsAg)-positive); or severe intercurrent illness.

Participants were allowed to take proton pump inhibitors and other gastric acid modifiers during the study period if medically necessary but were recommended to not take these medications during the PK sampling visits.

### 2.4. PK Assessments

Women underwent PK sampling at 25 + 0 to 26 + 6 weeks’ (Visit 1), 29 + 0 to 31 + 6 weeks’ (Visit 2), and 33 + 0 to 34 + 6 weeks’ gestation (Visit 3). These visits were performed following an 8 h overnight fast (nothing but water). However, in the event food or beverages other than water had been consumed in the 8 h preceding study drug administration on the day of the intensive PK visit, the type and amount of food and beverage was recorded. The timing of the last SOF/VEL dose was also carefully recorded.

A pre-dose blood sample was obtained, followed by a standardised breakfast, observed dosing of SOF/VEL, and blood samples collected at 0.5, 1, 1.5, 2, 4, 5, and 24 h post dose. Participants had the option to participate in a longer, more intensive PK visit schedule which required collection of additional samples at 6 and 8 h post dose. For the first four hours post study drug administration, participants were asked to record all foods and beverages consumed other than water, but there were no restrictions after this time frame.

### 2.5. Analytical Methods and PK Analysis

PK blood samples were processed for plasma and analysed using a validated liquid chromatography–tandem mass spectrometry (LC-MS/MS) method to quantify SOF, GS-331007, and VEL [6]. The linear range for each analyte ranged from 5.00 ng/mL to 1500 ng/mL, and each analyte had a lower limit of quantitation (LLOQ) of 5.00 ng/mL.

Plasma concentrations of SOF, GS-331007, and VEL were analysed by noncompartmental analysis (Phoenix Certara, version 8.4). The following PK parameters are reported for each analyte, unless indicated otherwise: maximum plasma concentration (C_max_), the predicted plasma concentration at 24 h post dose (C_tau_), area under the curve over the 24 h dosing interval (AUC_tau_) or through the last measurable time point (AUC_last_), half-life (t_1/2_), apparent volume of distribution (V/F), and apparent oral clearance (CL/F).

AUC_tau_ and AUC_last_ were calculated using linear up-log down trapezoidal rule. Concentrations below the limit of quantitation (BLQ) were set to 0 when they occurred before C_max_, and when they occurred after C_max_, the first observation was set to half the LLOQ and then remaining time points were treated as missing. C_max_ was based on direct observation of the data. C_tau_ was based on the measured concentration for samples collected exactly at 24 h post dose or predicted concentrations at 24 h based on partial AUC estimates. The elimination half-life was calculated as 0.693 divided by the elimination rate constant. V/F was calculated as CL/F divided by the elimination rate constant, and CL/F was calculated as the dose of SOF (400 mg) or VEL (100 mg) divided by the AUC.

### 2.6. Efficacy and Safety Assessment

Maternal HCV RNA was measured at the end of treatment and at 12 weeks after the end of treatment to evaluate the efficacy of SOF/VEL. HCV cure was defined as sustained viral response at 12 weeks after completion of therapy (SVR12). Neonatal HCV infection was evaluated by an HCV RNA test at ≥6 months of age. To assess the safety of SOF/VEL use during pregnancy, adverse obstetric events occurring during treatment and any major neonatal malformations were recorded.

### 2.7. Acceptability Assessment

Participants completed an investigator-designed questionnaire to determine their attitude to and acceptability of SOF/VEL treatment during pregnancy.

### 2.8. Statistical Analysis

Baseline demographics, PK, safety, and efficacy outcomes were summarised using descriptive statistics. The primary outcome was to determine if the PKs of SOF and VEL were similar in pregnancy compared to those in non-pregnant women. Linear mixed effects modelling was performed to determine if AUC_tau_, C_max_, and C_tau_ differed across PK visit for each analyte, and to allow for a single geometric mean to be calculated across the repeated measures within participants (R Version 4.4.1). AUC_tau_, C_max_, and C_tau_ during pregnancy were then compared to historical data in non-pregnant women to generate geometric mean ratios with 90% confidence intervals (CIs).

## 3. Results

### 3.1. Participant Characteristics

Five participants were recruited to the study. The first participant was enrolled in December 2019, and the last participant was enrolled in January 2023. Enrolment was affected by the COVID-19 pandemic, and subsequently the study was halted in 2024 due to slow recruitment. Baseline demographic and clinical characteristics are summarised in Table 1. The majority of participants were recently diagnosed with hepatitis C and none were on gastric acid-modifying medications.

### 3.2. Pharmacokinetic Outcomes

PK sampling was performed in all five participants across all three study visits. Four participants on PK Visits 1 and 2 and three participants on PK Visit 3 underwent intensive PK assessments with additional sampling. For two participants on PK Visit 2 and one participant on PK Visit 3, elevated concentrations at the 24 h post-dose sample were noted for GS-331007 and VEL, suggesting a dose was taken prior to collection of this sample. In these cases, concentrations at the 24 h post-dose time point were replaced with the pre-dose (time 0) concentration to facilitate more accurate estimation of the PK parameters.

**Figure 1:**
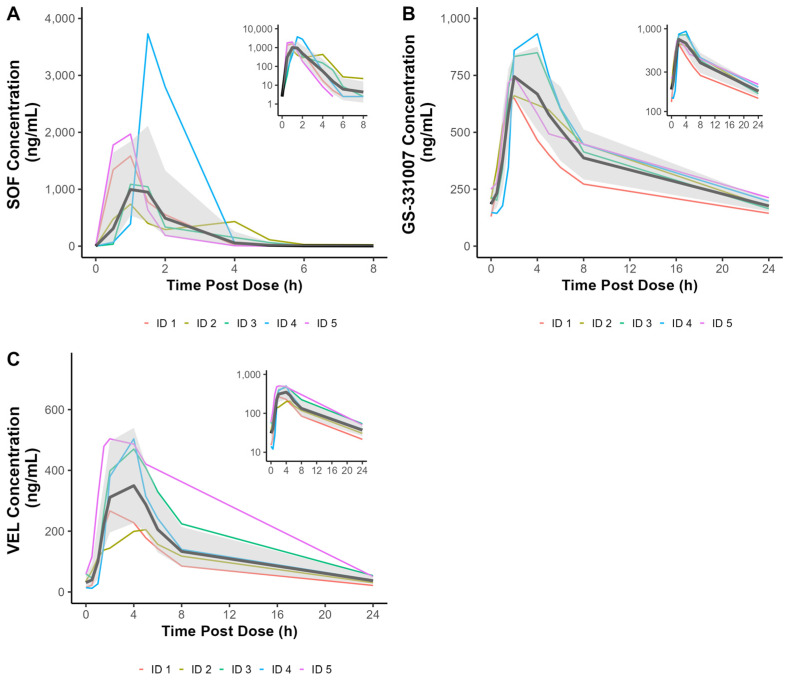
Plasma concentration–time curves for (**A**) sofosbuvir (SOF), (**B**) GS-331007, and (**C**) velpatasvir (VEL). Representative results displayed for PK Visit 1. The black line represents the geometric mean at each nominal time point with the 90% confidence interval shaded in gray.

**Figure 2:**
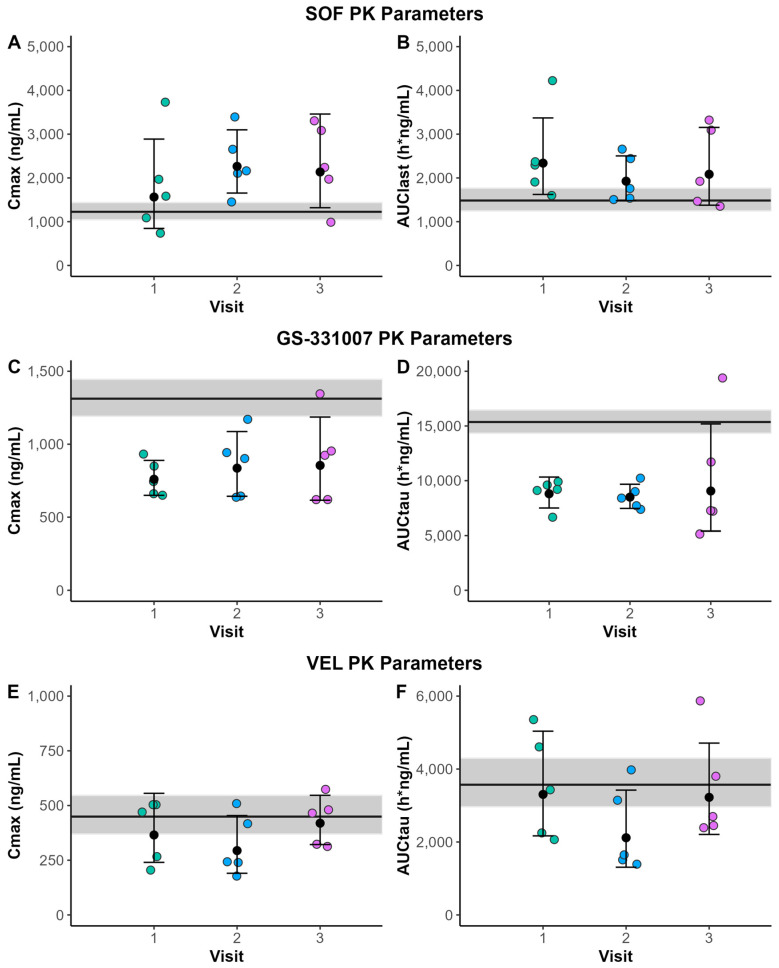
PK parameters at PK Visits 1, 2, and 3 for (**A**) sofosbuvir (SOF) Cmax, (**B**) SOF AUClast, (**C**) GS-331007 Cmax, (**D**) GS-331007 AUCtau, (**E**) velpatasvir (VEL) Cmax, and (**F**) VEL AUCtau. Data are presented as geometric mean and geometric standard deviation. The grey line represents the geometric mean with 90% CI from non-pregnant historical controls. Due to the short half-life of SOF and inability to calculate an accurate elimination rate, AUC_last_ is reported.

Concentration–time curves for each analyte are presented in Figure 1, and PK parameters during pregnancy are presented in Table 2. PK parameters did not differ by study visit, and thus the results reflect the geometric mean from the mixed model analyses. When compared to non-pregnant women, SOF AUC_last_ and C_max_ were 42.2% (90% CI 3.5%, 95.4%) and 59.8% (90% CI 7.5%, 138%) higher in pregnancy, respectively (Figure 2). In contrast, the AUC_tau_ and C_max_ of GS-331007, the inactive metabolite of SOF, were 42.8% (90% CI −54.7%, −27.9%) and 37.8% lower (90% CI −49.5%, −23.4%) in pregnancy, respectively. The AUC_tau_ and C_max_ for VEL were comparable between pregnant and non-pregnant women (−20.7% [90% CI −46.5%, 17.5%] and −20.8% [90% CI −44.8%, 13.6%], respectively) while C_tau_ was 41.5% (90% CI −64.6%, 3.4%) lower in pregnancy.

### 3.3. Efficacy and Safety Outcomes

Birth outcomes and HCV nucleic testing (PCR) results are summarised in Table 3. All infants were delivered at term (≥37 weeks), and no severe neonatal complications were reported at birth. Two (40%) infants had a negative HCV PCR test at 6 months; the other three infants were lost to follow-up. Maternal HCV PCR tests were negative at the end of treatment in four (80%) participants, and at 12 weeks after treatment completion in five (100%) participants; one participant at end of treatment did not have test results available.

### 3.4. Acceptability Outcomes

The main reasons for study participation were to be cured (75%), to protect the baby (50%), and to help others (25%) among the four participants who responded to this question. Two of five participants reported seeing a specialist prior to pregnancy, 80% reported discussing the study with family/friends prior to consent, and 80% reported their family/friends were supportive of participating in the study. SOF/VEL was generally well tolerated, with no significant side effects reported.

## 4. Discussion

In the present study, SOF AUC_last_ and C_max_ were higher, whereas GS-331007 AUC_tau_ and C_max_ estimates were lower in pregnancy. VEL AUC_tau_ and C_max_ were comparable to non-pregnant women, whereas C_tau_ was slightly reduced. Despite these differences, 4/5 participants achieved SVR12 (with one participant achieving an end of treatment negative PCR but did not attend for follow-up testing at 12 weeks post completion of treatment). SOF/VEL was generally well tolerated, with no significant side effects reported.

The PK results in this study largely mirrored the previously reported results of SOF/VEL use in pregnancy from Chappell et al. (2024) [6]. The changes in exposure to SOF (~40% increase) and GS-331007 (~40% decrease) in the present study were comparable to previous reports [6]. In both the present study and previous results, VEL AUC_tau_ and Cmax were similar between pregnant and non-pregnant women, but we identified lower C_tau_ values.

Numerous physiological changes occur in pregnancy that can affect drug PK [8]. Increases in gastric pH (becomes less acidic) during pregnancy may contribute to the 20% reduction in systemic exposure to VEL since the solubility of VEL decreases with increasing pH [9]. None of the participants were prescribed gastric acid modifying medications. Furthermore, the slightly reduced VEL exposure in this study could also be due to increases in CYP3A4 activity during pregnancy, which is a minor metabolising enzyme of VEL [9,10]. CYP2B6 and CYP2C8 also play a minor role in VEL metabolism; however, changes in these enzymes during pregnancy less characterised.

Increased SOF exposure in pregnancy may be due to multiple pregnancy-related physiological changes. These include delayed gastric emptying, reduced intestinal motility, and increased intestinal blood flow [7], all of which may increase oral absorption of SOF. Decreased expression of carboxylesterase 1 (CES1) is also possible, but there are conflicting reports on CES1 expression and activity in the presence of pregnancy-related hormones. Wu et al. [11] found that 17β-estradiol reduced both expression and activity of CES1 in human and mouse hepatocytes, while Fashe et al. [12] reported increased CES1 protein concentrations in human hepatocytes in the presence of pregnancy-related hormones. A separate clinical PK study, however, identified no differences in the PK of oseltamivir, another CES1 substrate, during pregnancy, but the renally eliminated carboxylate metabolite was significantly lower [13]. Further studies are needed to clarify CES1 changes in pregnancy. GS-331007 is a metabolite of sofosbuvir and is predominantly eliminated by glomerular filtration and tubular secretion [9,10]. The lower systemic exposure to GS-331007 in pregnancy in our study may be explained by increases in glomerular filtration rates.

Whilst PK changes were observed for SOF/VEL in this study, all five pregnant participants achieved either an end of treatment or 12-week post treatment negative HCV nucleic test, supporting the use of this regimen in pregnancy without dose adjustment. Our data are reassuring that across different settings and populations, similar PK results are found. Furthermore, the medication was well tolerated in pregnancy, as has been reported in pivotal phase three trials outside of pregnancy [13]. In terms of safety in pregnancy, this small study is inadequately powered to draw any conclusions, but no safety concerns were apparent.

There are several limitations of this study. Firstly, the small numbers recruited were fewer than originally planned. Recruitment was impacted by the COVID-19 pandemic as both antenatal sites converted to predominantly telehealth appointments rather than face to face. In addition, three of the five women did not attend arranged paediatric follow-up despite multiple attempts at contact and rescheduling of appointments. In relation to the PK analysis, a further limitation was that the AUC_tau_ values tended to be higher in participants without the additional samples.

These results add to the limited published experience of prescribing antivirals in pregnancy and provide further support for a larger ongoing prospective study [14] and other efforts to support HCV treatment in pregnancy [15]. SOF/VEL was safe and well tolerated. For two of the participants, the HCV was newly diagnosed based on antenatal screening. This highlights the unique opportunity in pregnancy where women are routinely tested and may be identified at an early stage in the disease prior to liver damage occurring. This may also be an opportune time for treatment given pregnant women are generally more engaged in their healthcare during pregnancy. When surveyed about motivations to participate in this study, most were driven by a desire to be cured and to protect the baby. This highlights additional drivers that may be leveraged in pregnancy to support adherence and completion of treatment.

## 5. Conclusions

In the present study, SOF AUC_tau_ and C_max_ were higher, and VEL exposures were slightly reduced. Despite these differences, treatment was effective in curing women in pregnancy with HCV and was well tolerated, with no significant side effects reported. This data provides further evidence supporting the use of antiviral therapy in pregnancy to not only cure pregnant women and prevent maternal HCV-related liver disease but also to help prevent perinatal transmission of HCV.

## Figures and Tables

**Table 1:** Baseline characteristics of participants.

Characteristic	Number (%)
Age, years median (IQR)	32 (23–34)
Country of Birth	
Australia	4 (80%)
Other	1 (20%)
Duration of HCV Diagnosis	
Diagnosed during current pregnancy	2 (40%)
<1 year	1 (20%)
1–5 years	1 (20%)
>5 years	1 (20%)
Mode of Infection	
Perinatal	2 (40%)
Injection Drug Use	2 (40%)
Unknown	1 (20%)
Reason for HCV Testing	
Antenatal Screen	3 (60%)
Contact Tracing	1 (20%)
Routine Health Check	1 (20%)

Continuous data are presented as median (IQR). Categorical data are presented as n (%).

**Table 2:** SOF, GS-331007, and VEL PK parameters in pregnant women.

Analyte	AUC_tau_ (h·ng/mL)	C_max_ (ng/mL)	C_tau_ (ng/mL)	t_1/2_ (h)	CL/F (L/h)	V/F (L)
SOF	2110 ^a^ (34.7)	1960 (50.9)	BLQ	0.44 (34.4)	181 (30.6)	117 (53.8)
007	8780 (30.6)	816 (24.9)	160 (59.2)	12.5 (44.7)		
VEL	2830 (47.4)	356 (39.8)	29.1 (69.4)	7.56 (46.8)	31.8 (38.6)	349 (64.7)

Values presented as geometric mean (%CV) from participants accounting for repeated measures across 3 PK visits. Abbr: BLQ = below limit of quantitation; SOF = sofosbuvir; 007 = GS-331007; VEL = velpatasvir. ^a^ Due to the short half-life of SOF and inability to calculate terminal elimination rate in some participants, AUC_last_ is reported.

**Table 3:** Birth outcomes and HCV RNA post treatment.

Outcome	
Gestation at birth (weeks)	38 (38–39)
Birth weight (g)	3040 (2715–3415)
End of treatment (EOT) HCV PCR	
Negative	4 (80%)
Not tested	1 (20%)
12-week post EOT HCV PCR	
Negative	5 (100%)
6-month Infant HCV PCR	
Negative	2 (40%)
Lost to Follow-up	3 (60%)
Maternal Complication	
Post-partum haemorrhage	1 (20%)
3rd-degree perineal tear	1 (20%)
None	3 (60%)
Neonatal Complication	
Partial fusion 4th and 5th digit	1 (20%)
None	4 (80%)
Apgar Score 9,9	5 (100%)

## Data Availability

Data for this research is unavailable due to privacy restrictions.
